# Plasma prolactin in patients with colorectal cancer

**DOI:** 10.1186/1471-2407-4-97

**Published:** 2004-12-23

**Authors:** Ahmad Reza Soroush, Hosein Mahmood zadeh, Mehrnush Moemeni, Behnam Shakiba, Sara Elmi

**Affiliations:** 1 Department of surgery, Tehran University of Medical Sciences, Shariati hospital, kargar shomali st, Tehran 14114, Iran; 2 Research Development Centre, Tehran University of Medical Sciences, Shariati hospital, kargar shomali st, Tehran 14114, Iran; 3 Students' Scientific Research Centre (SSRC), Tehran University of Medical Sciences, Tehran, Iran

## Abstract

**Background:**

Colorectal cancer is a common malignancy of the gastrointestinal tract. It is the second cancer cause of death in females and third in males. Production of prolactin has been reported with several tumours. However, elevated prolactin plasma levels in colorectal cancer patients remained unclear.

**Methods:**

In this cross sectional study serum prolactin and carcinoembryonic antigen (CEA) concentrations were assayed using immunoradiometric assay kits, preoperatively in 47 patients, and the results were compared with 51 age and sex matched controls.

**Results:**

Prolactin and CEA concentration in patients were significantly more as compared with controls. Hyperprolactinemia was found in 36 (76.6%) patients, while 28 (59.6%) had high level of CEA.

**Conclusions:**

Prolactin may be a better tumour marker than CEA in patients with colorectal malignancy.

## Background

Colorectal cancer is the third highest cause of cancer mortality [1]. In Australia, the United Kingdom and the United States, it is the second common cancer for women after breast cancer (age-standardised incidence 22–33 per 100,000), and men after prostate or lung cancer (age-standardised incidence 31–47 per 100,000). About 148,300 new cases are reported each year in the USA and 56,600 Americans die annually from this cause [2]. Prognostic factors are very important for the evaluation, judgment and optimal treatment of patients with cancer.

Carcinoembryonic antigen (CEA) has been recognized as a serum marker for colorectal cancer in the past three decades [3] and has an important role in the management of colorectal cancer [4].

Prolactin (PRL) is a hormone with multiple biological actions, synthesized by the anterior pituitary gland [5] and is best known for its roles in the mammary gland. However, it is now revealed that PRL is able to exert its effects on additional cells and tissues (decidual cells of the placenta, bone, brain, lymphocytes and breast epithelial cells) [6,7]. PRL is secreted not only by lactotrophic cells of the pituitary gland but also by a variety of other normal tissues and human tumours [8] including malignant tumours of the lung[9], kidney[10], uterine[11], ovary [12], and breast[13]. Increase of PRL in colorectal cancer is unclear. Bhatavdekar, et al reported a significantly higher preoperative prolactin levels in patients with colorectal carcinoma [14], While, Indinnimeo et al and Carlson et al could not confirm hyperprolactinemia in patients with colorectal cancer. [15,16] These conflicting results do not support the hypothesis of increasing level of PRL in colorectal carcinoma.

The aim of this study was to see whether PRL levels are increased in colorectal cancer and to compare the preoperative serum levels of PRL and CEA concentrations in colorectal cancer patients; also we intended to find the possible correlation between hyperprolactinemia and the stage of colorectal cancer.

## Methods

This cross-sectional study included 47 patients with colorectal cancer (18 women, 29 men; mean age 55.4 years, range 32–81) and 51 non-cancer patients (21 women, 30 men; mean age 58.5 years, range 20–81), who were candidates of colectomy. All patients were admitted in Shariati general hospital affiliated to Tehran University of Medical Sciences (TUMS) from April 2001 to January 2003. All women in the study were postmenopausal. None of the patients were on any drugs known to increase plasma prolactin levels in the last 6 months prior to the study and were not affected by endocrine, renal, and psychiatric disorders. There was no history of rectal bleeding and family history of colorectal cancer. Peripheral venous blood samples were daily collected from seven days before the operation to measure serum PRL and CEA levels. Blood samples were collected between 07:30 a.m. and 09:30 a.m. to avoid diurnal variation of PRL. Serum prolactin levels were measured with immunoradiometric assay (IRMA) kits produced by Kavoshian co (Tehran-Iran). Serum CEA levels were assayed by IRMA, who used kits from IMMUNOTECH co (Marseille-France). The PRL and CEA assays were carried out within 10 days of sampling. The cut off levels for PRL and CEA were 20 ng/ml and 5 ng/ml serum respectively [17,18]. Stages of cancer were determined by pathology assessment of neoplastic specimens.

The study was approved by the Vice-Chancellor of ResearchEthic Committee of TUMS.

The data evaluated by Chi-square test and T test using the Statistical Package of Social Science (SPSS Inc., Chicago, IL) for Windows version 11.5. A P-value of <0.05 was considered statistically significant.

## Results

The number of male and female participants was 29 and 18 in case and 30 and 21 in control group.

We compared serum PRL and CEA level of 47 patients with 51 healthy controls. There was no significant difference between the age and the sex distribution of the patients and control groups.

Mean PRL serum level in colorectal cancer group was 21.6 ± 8.1versus 10.5 ± 4.6 in control group, which was significantly different (p < 0.0001). In other words 36 patients showed hyperprolctinemia (76.7%) while there was only one such patient in the control group (1.9%).

CEA level in 59.6% of the cancer group was above the normal compared with 1.9% of controls (P < 0.0001).

**Figure 1:**
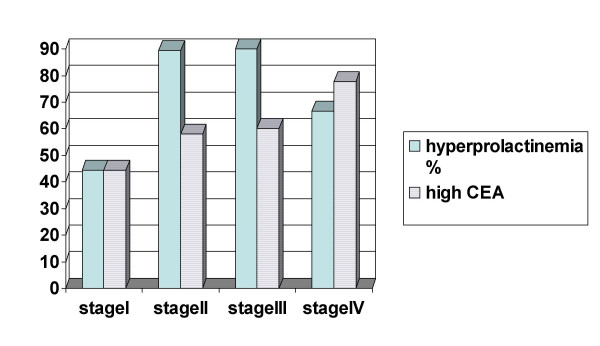
Hyperprolactinemia and high CEA levels in stages of colorectal cancer.

Out of 47 patients in the cancer group 9 were in stage I (19.1%), 19 in stage II (40.4%), 10 in stage III (21.3 %) and 9 in stage IV (19.1%). Hyperprolactinemia and high CEA concentrations were found in all stages (figure 1).

## Discussion

Prognostic factors are crucial for the evaluation and optimal treatment of patients with cancer [17]. Therefore tumour markers have a greater role in the assessment of therapeutic response. Measurements of serum tumour markers may be helpful in detecting the metastatic process while still in the sub clinical phase [15]. The ectopic secretion of hormones like PRL by non-endocrine neoplasms is well recognized and used as therapeutic monitoring [15].

The main source of PRL is the pituitary gland, but in recent years, some studies have reported hyperprolactinemia in patients with breast [13], lung [9], prostate and ovary tumours [12].

Elevated circulatory levels of this hormone have also been detected in colorectal cancer. Bhatavdekar et al reported a high preoperative serum concentration of PRL in patients with colorectal cancer [14]. Ilan et al. reported elevated levels of PRL in 53% of the patients with colorectal malignancy [19]. Baert et al, in contrast didn't determine preoperative hyperprolactinemia in a series of 32 patients and their study didn't support the hypothesis of ectopic prolactin production by colorectal cancer [20]. Indinnimeo, et al found no hyperprolactinemia and prolactin positive immunostaining in colorectal cancer [15]. Previous studies, that reported normal levels of PRL [20,15], have suggested such factors as renal, endocrine and psychiatric disorders, medications and premenopausal situation may be the cause of hyperprolactinemia in patients with colorectal neoplasms. In the present study, patients with above factors were excluded and our results confirmed preoperative hyperprolactinemia in colorectal cancer. We found no correlation between the plasma PRL concentration and the stage of the tumour.

CEA present in blood during fetal life, falls to very low levels in most adults, and circulates in high concentrations in patients with some cancers [21]. CEA is over expressed in most colorectal cancers and is an important tumour marker in the management of colorectal cancer. A preoperative high CEA value suggests advanced disease either locally or with a distant metastasis. The preoperative serum CEA level can be a useful predicting factor regarding the outcome of the surgical operation [4]. In the present study we found that serum PRL and CEA levels are increased in patients with colorectal cancer but the greater portion of the patients had an increased level of PRL compared with elevated level of CEA. Considering the fact that laboratory cost for detecting CEA is higher than PRL, We suggest PRL as a valuable tumour marker in colorectal cancer. There are studies that support the tumour marker value of PRL [18,22] and studies that do not [16,20].

## Conclusions

This study demonstrates that PRL, a pituitary gland hormone, is elevated in colorectal cancer. Moreover, our results show that PRL may be a better tumour marker than CEA in patients with colorectal malignancy. However, further prospective studies are warranted to better understand the evaluation of PRL as a prognostic factor, and to follow up patients after operation to determine the possible correlation between PRL levels, survival and response to therapy.

## Competing interests

The author(s) declare that they have no competing interests.

## Authors' contributions

Dr Ahmad Reza Soroush and Dr Hosein Mahmood zadeh participated in the design of study, acquisition of data and interpretation of data. Dr Mehrnush Moemeni participated in the design of study, acquisition of data, interpretation of data, and drafting the article. Behnam Shakiba participated in acquisition of data, interpretation of data, drafting the article and final approval of this version. Sara Elmi participated in analysis and interpretation of data, drafting the article and final approval of this version. All authors read and approved the final manuscript.

## Pre-publication history

The pre-publication history for this paper can be accessed here:
